# The role of infections and coinfections with newly identified and emerging respiratory viruses in children

**DOI:** 10.1186/1743-422X-9-247

**Published:** 2012-10-27

**Authors:** Maurizia Debiaggi, Filippo Canducci, Elisa Rita Ceresola, Massimo Clementi

**Affiliations:** 1 Dipartimento di Scienze Clinico-Chirurgiche, Diagnostiche e Pediatriche, sezione di Microbiologia, 20132, Milan, Italy; 2 Laboratorio di Virologia e Microbiologia, Università degli studi dell'Insubria, Istituto Scientifico San Raffaele, Via Olgettina 58, 20132, Milan, Italy

## Abstract

Acute respiratory infections are a major cause of morbidity in children both in developed and developing countries. A wide range of respiratory viruses, including respiratory syncytial virus (RSV), influenza A and B viruses, parainfluenza viruses (PIVs), adenovirus, rhinovirus (HRV), have repeatedly been detected in acute lower respiratory tract infections (LRTI) in children in the past decades. However, in the last ten years thanks to progress in molecular technologies, newly discovered viruses have been identified including human Metapneumovirus (hMPV), coronaviruses NL63 (HcoV-NL63) and HKU1 (HcoV-HKU1), human Bocavirus (HBoV), new enterovirus (HEV), parechovirus (HpeV) and rhinovirus (HRV) strains, polyomaviruses WU (WUPyV) and KI (KIPyV) and the pandemic H1N1v influenza A virus. These discoveries have heavily modified previous knowledge on respiratory infections mainly highlighting that pediatric population is exposed to a variety of viruses with similar seasonal patterns. In this context establishing a causal link between a newly identified virus and the disease as well as an association between mixed infections and an increase in disease severity can be challenging. This review will present an overview of newly recognized as well as the main emerging respiratory viruses and seek to focus on the their contribution to infection and co-infection in LRTIs in childhood.

## Introduction

In the last decade thanks to progress in molecular technologies, newly discovered viruses have been identified. In 2001 human Metapneumovirus (hMPV) was identified, followed by discoveries or emergence of other respiratory viruses or virus strains: coronaviruses NL63 (HcoV-NL63) and HKU1 (HcoV-HKU1), human Bocavirus (HBoV), new enterovirus (HEV), parechovirus (HpeV) and rhinovirus (HRV) strains, polyomaviruses WU (WUPyV) and KI (KIPyV) and the pandemic H1N1v influenza A virus [1-8].

These discoveries have heavily modified previous knowledge on respiratory infections and have now highlighted that although until recently most viral lower respiratory infections in infants and young children were attributed to respiratory syncytial virus (RSV), PIVs, adenovirus, HRV and influenza viruses, pediatric population is indeed exposed to a variety of other viruses with a similar seasonal pattern [9-11]. In this context, establishing a causal link between a newly identified virus and the disease as well as an association between mixed infections and an increase in disease severity can be challenging. This review will present a brief overview of newly recognized as well as the main emerging respiratory viruses and seek to focus on the their contribution to infection and co-infection in LRTIs in childhood.

### Metapneumovirus

Metapneumovirus was first recognized in 2001 in the Netherlands from nasopharyngeal aspirates collected during a 20-year period in 28 hospitalized children and infants with acute respiratory tract infection (RTI) having signs and symptoms similar to that of RSV infection [4]. The virus genomic sequence was identified by using a randomly primed PCR protocol and revealed to be closely related to the avian pneumovirus, a member of the *Metapneumovirus* genus, in the Paramixoviridae family, Initial studies following the first hMPV identification indicate that it causes upper and lower RTIs in patients of all ages, but mostly in children aged below 5 years [12-17]. A large epidemiological retrospective study examined nasal washes collected over a 20-year period during acute respiratory illnesses in an outpatient cohort of children [18]. Over the entire study period, hMPV was detected in 1%-5% of pediatric upper RTIs (UTRIs), with variation from year to year. Several reports indicate that hMPV is a commonly identified cause of pediatric lower RTIs, and is second only to RSV as cause of bronchiolitis in early childhood [16,19-25]. While bronchiolitis, is the most common presentation of hMPV illness, other reported syndromes have included asthma exacerbation, otitis media, flulike illness, and community-acquired pneumonia [12,15,26-29]. Several studies have found hMPV –RSV co-infection rates of approximately 5-14% [30-34]. Nevertheless, in a study conducted in the Netherlands in children admitted to hospital for lower RTIs (LRTIs), no virus co-infection between RSV and hMPV was detected [35]. Different controversial reports suggest an association between RSV-hMPV coinfection and an increase in the disease severity or the absence of an association between dual infection and disease severity. Greensill and colleagues [36] reported a 70% rate of co-infection with hMPV in a cohort of infants with critical RSV bronchiolitis who required intensive care in the United Kingdom, suggesting that dual infection with RSV and hMPV may predispose for a more severe disease. In another study from the United Kingdom, hMPV and RSV co-infection was associated with increased disease severity and higher risk of admission to the pediatric intensive care unit [37]. Similar findings are supported by other studies suggesting that in young children, coinfections with RSV and hMPV are more severe than infections with either RSV or hMPV alone, requiring a longer hospitalization and supplemental oxygenation [38,39]. However, such synergistic association has not been found in other population-based and case–control studies of hospitalized children [16,33-35,40-42]. In particular, two studies evaluated the epidemiology of hMPV coinfection in children with LRTI caused by RSV and demonstrated no hMPV and RSV co-infection in mechanically ventilated children suggesting that co-infection with hMPV is not associated with a more severe course of RSV-LRTI [35,42]. In addition, in a prospective 2-year study in hospitalized infants with acute respiratory diseases, the role of RSV as a major respiratory pathogen was not influenced by the co-circulation of other emerging viral agents with similar seasonal distribution. In particular, RSV-hMPVs co-infections were significantly observed in less severe respiratory disease when compared to unique RSV infections [34]. The possible synergistic interaction between hMPV and the severe acute respiratory syndrome (SARS) coronavirus was also suggested during the 2003 SARS outbreak in Hong Kong and Canada [43,44]. In one case report, in an infant with SARS CoV infection, fatal encephalitis was correlated with hMPV infection as hMPV RNA was detected post-mortem in brain and lung tissue [26]. Nevertheless, in experimental studies performed in macaques, a synergy between hMPV and SARS was not confirmed [45]. In addition, infections of hMPV with respiratory viruses different from RSV, have also been occasionally reported but no sufficient data are available to discuss epidemiology or association with clinical disease presentation [34,46,47] (Table 1).

**Table 1:** Recent epidemiological papers on respiratory infections and co-infections

**PAPER**	**VIRUSES ANALYSED (detection methods)**																	**POPULATION**	**CONCLUSIONS**
	**Flu**			**HRV**	**HEV**	**HPeV**	**PIV**	**hMPV**	**ADV**	**RSV**	**Coronavirus**				**HBoV**	**Polyomavirus**			
	**H1N1**	**A**	**B**								**HCoV 229E**	**HCoV OC43**	**HCoV NL63**	**HCoV HKU1**		**WUPyV**	**KIPyV**		
Greensill, 2003								**X(a)**		X(a)								pediatric (30, ventilated and with bronchiolitis)	70% rates of co-infection RSV-hMPV in a cohort of infants with bronchiolitis, suggesting that dual infection may predispose for a more severe disease.
Viazov, 2003								**X(a)**		(d)								children <2 years (63) with RTD	17,5% hMPV positive, 23% RSV positive, 4.7% hMPV-RSV coinfections. Similar symptoms between hMPV+ and RSV+ children.
Esper, 2004								**X(a)**										children <5 years (688) negative for RSV, PIV, Flu A and B, ADV	8% hMPV positive samples
Xepapadaki, 2004		X(a)	X(a)	X(a)			X(a)	**X(a)**	X(a)	X(a)	X(a)	X(a)	X(a)	X(a)				pediatric (56) with acute bronchiolitis	16% bronchiolitis is hMPV positive, 67.9% RSV positive, without clinical d(d)ference.
Konig, 2004								**X(a)**		X(a)								children requiring intensive support (85)	coinfections with RSV and hMPV are more severe than infections with either RSV or hMPV alone in young children
Semple, 2005								**X(a)**		X(a)								<2-year-old infants with bronchiolitis(196)	hMPV and RSV co-infection is associated with increased disease severity
van Woensel, 2006								**X(b)**		X(b)								pediatric (30, mean age 10 weeks, ventilated and with bronchiolitis)	No virus co-infection between RSV and hMPV in a cohort of infants with bronchiolitis
Foulongne, 2006		(d)	(d)				(d)	**X(a)**	(d)	(d)								589 children hospitalized with respiratory disease<5 years	8.5% rates of hMPV infections, the second leading cause of RTD after RSV, 30% of the cases are hMPV-RSV coinfections. The duration of hospitalization and requirement for supplemental oxygen were increased in case of hMPV-RSV coinfections
Lazar, 2004								**X(a)**		X(a)								46 children with mild to severe RSV disease (PIV, fluA and B, ADVnegative)	hMPV did not contribute to the severity of RSV disease
Canducci, 2008								**X(a)**		X(a)	X(a)	X(a)	X(a)	X(a)	X(a)			322 infant patients with acute respiratory disease	RSV-hMPVs co-infections were observed in less severe respiratory disease when compared to RSV mono-infections
Chiu, 2005		(d)	(d)				(d)	X(a)	(d)	(d)	**X(a)**	**X(a)**	**X(b)**					hospitalized with fever and acute respiratory symptoms (587)	4.4% HCoV infections. HCoV-NL63 can present as croup, asthma exacerbation, febrile seizures, and high fever.
van der Hoek,2006		X(a)	X(a)				X(a)			X(a)			**X(b)**					children<3 years with LRTIs (940)	HCoV-NL63 RNA was detected in 5.2% of cases;43% of the HCoV-NL63-positive patients with high viral load and absence of co-infection suffered from croup. Most co-infections were with RSV-A HCoV-NL63 co-infection with RSV-A occurred mainly in hospitalised patients in contrast to HCoV-NL63 co-infections with PIV3 that were exclusively present in the outpatient group. Lower HCoV-NL63 viral load in patients coinfected with RSV or PIV3 than in patients infected with HCoV-NL63 alone
Dare, 2007											**X(b)**	**X(b)**	**X(b)**	**X(b)**				1156 patients with pneumonia, 513 outpatients, 281 controls	1.8% of patients with pneumonia, 2.3% of outpatients and 2.1% of controls had HCoV infections. In control patients, infection with any HCoV type or with all types combined was not associated with pneumonia
Kuypers, 2007		X(a)					X(a)	X(a)	X(a)	X(a)	**X(b)**	**X(b)**	**X(b)**	**X(b)**				1043 pediatric (0–19 years old) respiratory specimens	CoVs were detected in 6.3% of specimens. 45.5% CoV-positive specimens also had another respiratory virus detected, most commonly RSV (67%). CoV subtypes NL63 and HKU1 accounted for the majority of CoVs detected.
Minosse, 2008				X(a)					X(a)				**X(a)**					hospitalized adult patients (433, mean age 56 years)	2% Hcov NL63 positive, 33% coinfected NL63-HRV, 10% coinfected NL63-ADV
Wu, 2008								X	X	X			**X(b)**					539 children < 15 years with respiratory disease	1.3% HCoV NL63 positive, 43% of HCoV infection are coinfections (RSV, ADV, hMPV)
Gaunt, 2010		X(b)	X(b)				X(b)		X(b)	X(b)	**X(b)**	**X(b)**	**X(b)**	**X(b)**				11661 respiratory samples	high rate of coinfections observed for HKU1, NL63 and OC43, mostly with RSV. No d(d)ferences of HCoV viral load were observed between single infection and RSV coinfection. Detection of CoVs should not be interpreted as representing an incidental infection without contribute to disease.
Choi, 2006		X(a) and (d)	X(a) and (d)	X(a)			X(a) and (d)	X(a)	X(a) and (d)	X(a) and (d)	X(a)	X(a)	X(a)		**X(a)**			515 children< 5 years old with LRTIs	The prevalence of HboV was the second (11%), the first was RSV (23%). Among HBoV infections, a high rate (38%) was coinfection.
Allander, 2007		X(b), (c), (d), (e)	X(b), (c), (d), (e)	(c), (e), X(a)	(c), (e), X(a)		X(b), (c), (d), (e)	(c), X(a)	X(b), (d), (e)	(c), (d), (e), X(a)	X(a)	X(a)	X(b), (e)	X(b), (e)	**X(b), (e)**			259 children (median age, 1.6 years) who had been hospitalized for acute expiratory wheezing	Acute HBoV infections appears associated with presence of viral DNA in the blood as the HBoV DNA was reported more prevalent in the patients blood during the acute symptoms than after recovery. High load and viremic HBoV infection were associated with respiratory tract symptoms, while detection of a low viral load in the nasopharinx alone has uncertain relevance
Christensen, 2008		X(b)	X(b)	X(b)	X(b)		X(b)	X(b)	X(b)	X(b)	X(b)	X(b)	X(b)		**X(b)**				HBoV was detected in 12% of samples. It was the fourth most common virus in the material after RSV (25%), HRV (17%) and hMPV(14%). Multiple viral infections were common and were present in 78% of the samples, more commonly RSV
Dina, 2009		X(a)	X(a)	X(a)			X(a)	X(a)	(d), (c), X(b), x(a)						**X(b), X(a)**			842 patients hospitalized with respiratory symptoms (mean age 22 years)	The prevalence of HBoV infection was 3.8%. HBoV. Viral load appears to be linked to the severity of the disease.
Wang, 2010		X(a)	X(a)	X(a)			X(a)	X(a)	X(a)	X(a)	X(a)	X(a)	X(a)	X(a)	**X(a),X(b), (e)**			817 children with respiratory tract infection	HBoV was ident(d)ied in 12% of samples. Co-infection rate with other respiratory viruses was 51%. HBoV was found frequently in children with respiratory tract symptoms associated with other viruses, and persisted in the respiratory tract, in serum and urine. The presence of IgM was significantly more prevalent in viremic patients and those diagnosed with high load of HBoV DNAin nasal/throat swabs
Don, 2010		(e)	(e)				(e)	(e)	(e)	(e)					**(e)**			124 children with presumptive pneumonia<15 years	Mixed infections were found in 25% of cases. Serological evidence of acute HBoV infection was found in 12% of children with pneumonia and in more than half of cases with single HBoV infection.This suggests that HBoV may be a fairly common cause of pneumonia in children
Soderlund-Venermo, 2009		X(a)	X(a)	X(a)	X(a)		X(a)	X(a)	X(a)	X(a)	X(a)	X(a)	X(a)	X(a)	**X(a), (e), X (b)**			259 weezing children <15 years, 115 healthy adults	Serologically confirmed primary HBoV infections detected in symptomatic children with no signs of other respiratory virus infections demonstrate that HBoV is a cause of acute wheezing in young children. Accurate HBoV diagnosis requires serologic analysis or PCR of serum, PCR of NPAs alone is insufficient. HBoV is the most probable cause of respiratory tract disease (d) the patients has single infection, a high viral load in NPA nasopharyngeal aspirates and viremia
Martin, 2010		X(a)	X(a)	X(a)			X(a)	X(a)	X(a)	X(a)	X(a)	X(a)	X(a)	X(a)	**X(b)**			119 children attending daycare	28% tested positive for HBoV. HBoV was detected significantly more often than any of the 14 respiratory viruses but HRVs. HboV DNA can persist for several months in the respiratory tract
Jin, 2012		X(a)	X(a)	X(a)			X(a)	X(a)	X(a)	X(a)			X(a)	X(a)	**X(a)**			813 children<14 years with acute lower respiratory tract infections	The most frequently detected virus was RSV (40.%), followed by HRV (20%), HBoV (11.5%), PIV1-3 (8%), AdV (7.5%), FluA (7%), HMPV (6.%), NL63 (4%), HKU1 (2.%) and FluB (0.98%). Of the HCoV-HKU1 and HCoV-NL63-positive samples, 74% were co-infected with at least another virus, most commonly HRV and RSV.
Esposito, 2012		X(a)	X(a)	X(b)	X(b)		X(a)	X(a)	X(a)	X(a)	X(a)	X(a)	X(a)	X(a)	**X(a)**			592 children with radiographically confirmed pneumonia	HBoV was the most frequently detected virus(10%) after RSV (31%) and HRV (24%)
Le, 2007		X(a)	X(a)	X(a)			X(a)	X(a)	X(a)		X(a)	X(a)	X(a)	X(a)		**X(b)**		2,263 samples from children <4 years of age and 374 from children >4 years of age for routine respiratory virus detection	2.7% samples positive for WU polyomavirus and 71% coinfected with other viruses. WU polyomavirus was the sole virus detected in 20 specimens from patients with respiratory illness, which suggests that it may be a respiratory pathogen. Repeated identification of WU polyomavirus in the same patients suggests that it may persistently infect humans
Han, 2007		X(a)	X(a)	X(a)			X(a)	X(a)	X(a)	X(a)	X(a)	X(a)	X(a)	X(a)	X(a)	**X(a)**		486 children with acute lower respiratory tract, 72 asymptomatic children<6 years	WUPyV was detected in 7% children with LARD, 4.2% of asymptomatic children and as coinfection with other respiratory viruses in 67.6%. Although WUPyV was frequently detected, its clinical role has not been distinguished from that of coinfecting viruses
Bialasiewicz, 2008		X(a)	X(a)	X(a)			X(a)	X(a)	X(a)	X(a)	X(a)	X(a)	X(a)	X(a)	X(b)	**X(b)**	**X(b)**	2866 respiratory sample from people with acute respiratory diseases (mean age 9.2 years)	KIV and WUV were found at a prevalence of 2.6% and 4.5%, respectively. Level of co-infection of KIV or WUV with other viruses was 74.7% and 79.7%, respectively. It is not possible to prove a causal relationship between the detection of KIV and WUV and respiratory disease from these findings
Neske, 2008		(d)	(d)				(d)		(d)						X(a)	**X(a)**		1,326 hospitalized children with acute respiratory diseases	4.9% positive WUPyV. 56% were co-infections with other viruses (ADV, fluA, hBoV and RSV).
Babakir-Mina, 2010																**X(b)**	**X(b)**	153 HIV-1-infected patients (mean age 42 years), 130 controls	2.6% KIPyV positive and 4.6% WUPyV positive among HIV-1–infected patients compared with 3.1% KIPyV positive and 0,8% WUPyV positive in blood donors. No association found between CD4+ cell counts in HIV-1 positive patients and infection with KIPyV or WUPyV
Debiaggi, 2010		X(a)	X(a)	X(a)			X(a)	X(a)	X(a)	X(a)	X(a)	X(a)	X(a)	X(a)	X(a)	**X(a)**	**X(a)**	31 asymptomatic adulthematopoietic stem cell transplant recipients;486 children with acute respiratory disease< 2 years	0.79% KIPyV, 0.79% WuPyV positive among transplant recipients; 1.4% KIPyV and 0.2% WUPyV in children. WU/KIPyVs have a low pathogenic potential in young children. Brief and asymptomatic infection can occur in hematopoietic transplant recipients.
Zhuang, 2011		X(b)	X(b)	X(b)			X(b)	X(b)	X(b)	X(b)					X(b)	**X(a)**		771 children with acute respiratory tract infection and 82 samples from healthy subjects	In most of infected children single WUPyV infection was detected.It suggests that the newly described polyomavirus can cause acute respiratory tract infection
Rao, 2011		X(a)	X(a)	X(a)			X(a)	X(a)	X(a)	X(a)	X(a)	X(a)	X(a)	X(a)		**X(b)**	**X(b)**	pediatric hematology or oncology patients and immunocompetent controls with acute respiratory illnesses	Prevalence of WUPyV and KIPyV is similar in hematology/oncology patients (3% and 5.6%, respectively) compared with the general pediatric population (5%and 2.3, respectively). High co-detection rates with other viruses (RSV and HRV) in both groups. Higher viral loads for KIPyV (but not for WUPyV) in the immunocompromised group was detected and infection with either virus occurred in older children compared with controls, which may suggest viral-reactivation
Lau, 2007		(d)	(d)	**X(a)**			(d)	X(a)	(d)	(d)	X(a)	X(a)	X(a)	X(a)				203 nasopharyngeal aspirates (NPAs), negative for common respiratory viruses from hospitalized children	HRV-C is an important cause of febrile wheeze and asthmatic exacerbations in children requiring hospitalization. No clear clinical difference has been noted between single or mixed HRV-C infections
Harvala, 2008		X(a)	X(a)			**X(a)**	X(a)	X(a)	X(a)	X(a)					X(a)			4,173 respiratory samples for routine respiratory virus detection	High rate of coinfections, ow frequency of detection and lack of clear disease associations indicate that HPeV1 and −6 are not major pathogens in individuals presenting with respiratory disease
Jin, 2009		X(a)	X(a)	**X(a)**			X(a)	X(a)	X(a)	X(a)	X(a)	X(a)	X(a)	X(a)	X(a)			406 children< 14 years with RTI	13% HRV positive (22% HRV-A, 12% HRV-B, 19% HRV-C). Monoinfection was observed in more than half of cases, HRV-C is an important cause of RTIs in children. Patients infected with HRV-C may exhibit different clinical features from patients infected with HRV-A/B
Miller, 2009				**X(a)**														1052 hospitalized children< 5 years with acute respiratory illness	HRVCs were detected in 7% of children hospitalized for fever or respiratory conditions and constituted almost half of all HRVs-associated hospitalizations, suggesting that this novel group causes a substantial burden of pediatric disease
Yozwiak, 2010					**X(a)**													3,800 children aged 2 to 13 years with respiratory illness	EV109 isolates were then detected in 1.6% of respiratory samples of children with influenza like illness (ILI) and recognized to have a pathogenetic role in the illness
Debiaggi, 2012		X(a)	X(a)	X(a)	X(a)		X(a)	X(a)	X(a)	X(a)	X(a)	X(a)	X(a)	X(a)	X(a)	X(a)	X(a)	1149 nasopharingeal aspirates	HEV109 infection may be associated to ARDs both in infants and in hematopoietic stem cell transplantation recipients
Piralla, 2012		X(a)	X(a)	X(a)	X(a)	**X(b)**	X(a)	X(a)	X(a)	X(a)	X(a)	X(a)	X(a)	X(a)	X(a)	X(a)	X(a)	3,525 patients with respiratory syndrome	The prevalence of HpeV is 0.4%. The most commonly identified HPeVs were HpeV1(58%) and HpeV3 (37%). Although not circulating at high frequency and unlikely to cause respiratory syndrome, HPeV was associated with severe clinical syndromes in a minority of newborns. The frequent association of HPeV with other respiratory viruses may indicate a less pathogenic role for HPeV compared to the other viruses
Renois, 2010	**X(a)**	X(a) and X(b)	X(a)	X(a)	X(a)		X(a)	X(a)	X(a)	X(a)	X(a)				X(a)			56 adults and 39 children visited for influenza-like illnesses	31% of H1N1 infections, 16% coinfected with HRV (60%) and RSV, CoV229E, HBoV (20%). No difference in disease severity between single and mixed infections
Casalegno, 2010	**X(b)**			X(b)														pediatric (2121, mean age 3.8 years)	The presence of HRV reduce the risk of H1N1 infection.
Schnepf, 2011	**X(b)**	X(a)	X(a)	X(a)			X(a)	X(a)	X(a)	X(a)	X(a)	X(a)	X(a)					in adult and paediatric patients with Influenza-like illness (413)	16% of H1N1 infections, 19% of them were co-infections (mainly HRV). Among 50% of non-H1N1 infections were HRV infections and increase of H1N1 cases was associated with rapid HRV infection decline

(a) qualitative molecular detection; (b) quantitative molecular detection; (c) cell culture; (d) Immune Fluorescence; (e) serology.

The pathogens analyzed and the methods used for detection as well as the population characteristics and the main conclusions are reported. The principal pathogen under evaluation is indicated in bold. Flu=influenza virus; HRV= human rhinovirus; HEV= human enterovirus; HpeV= human parechovirus; PIV= parainfluenza virus; hMPV=human metapneumovirus; ADV=adenovirus; RSV=respiratory syncytial virus.

### Coronaviruses

Following the discovery of SARS-CoV, other human coronaviruses, HCoV-NL63 and HCoV-HKU1, were identified and recognized to be common causes of community-acquired respiratory infections.

HCoV-NL63, a member of the group I coronaviruses, was first detected in 2004 in the Netherlands from a child with bronchiolitis by using a new method for virus discovery based on the cDNA-amplified restriction fragment−length polymorphism technique (cDNA-AFLP) [3].

HCoV-HKU1, a group II coronavirus, was first detected in Hong Kong in 2005 from an adult patient with chronic pulmonary disease [1]. All attempts to grow a virus from his respiratory secretions failed until recently [48], but coronavirus RNA was initially detected by RT-PCR using pol gene consensus primers.

Like other coronaviruses, NL63 and HKU1 can also be detected in individuals of all ages, including elderly patients with fatal outcome [49] and those with underlying diseases of the respiratory tract [1]. However most frequently, the newly discovered coronaviruses are reported in 7 to 12-month old children with both upper and lower RTIs [34,49-53]. In studies conducted in children hospitalized with RTIs in China, from 2.6% to 3.8% of patients were positive for HCoV-NL63 and, in addition to causing upper respiratory disease, HCoV-NL63 was found in croup, asthma exacerbation, febrile seizures, wheezing and high fever cases [54,55]. The occurrence of co-infection with NL63 and other respiratory viruses, including other human coronaviruses, RSV, PIV, influenza A and B viruses and hMPV has been reported [34,55-58]. In a large study from Germany evaluating children under 3 years of age with LRTIs, most co-infections were with RSV-A, probably because of the high percentage of RSV-A infections and an overlap in seasonality. In addition, double infection of NL63 with RSV-B, and with PIV3 occurred in a minority of cases. HCoV-NL63 co-infection with RSV-A occurred predominantly in the hospitalised patients in contrast to HCoV-NL63 co-infections with PIV3 that were exclusively present in the outpatient group [59].

Following the first identification, HKU1 was found in respiratory samples from elderly patients and children mainly with underlying diseases [1,33,52,60]. The most common symptoms are rhinorrhea, fever, and abdominal breath sounds [33], but pneumonia, bronchopneumonia, bronchiolitis, and acute asthma exacerbations were also described in children in China [61,62].

In a study aimed to evaluate the overall prevalence of 10 respiratory viruses in children with acute LRTIs in China from 2006 to 2009, 73.47% of the HCoV-HKU1 and HCoV-NL63-positive samples tested positive for at least one other virus, most commonly HRV and RSV [54]. Similar data describing a high rate of coinfection of coronaviruses with RSV has also been previously reported [63]. In a report from the UK both dual and single infections associated with respiratory outcomes were observed for HKU1 as well as for NL63 and OC43 coronaviruses [51]. In this study a high number of coinfections was observed for HKU1, NL63 as well as for OC43, mostly with RSV. Similar rates of lower and upper infections were observed in single HKU1 or OC43 infection compared with coinfection, whereas both URTI and LRTI were observed more frequently in single compared to mixed infection with NL63. No differences in clinical outcome were observed between single and dual infections with RSV and Coronaviruses NL63, HKU1 or OC43 indicating that RSV may presumably facilitate coronavirus infection without increasing disease severity. However, in the same study considering viral load data, a role of these coronaviruses in coinfections in respiratory disease was suggested. In fact no differences were observed when coronavirus load was evaluated in single infection and in RSV coinfection, indicating both that infection with another respiratory virus does not affect the ability of NL63, HKU1 or OC43 to establish infection and replicate, and that detection of coronaviruses in mixed infection should not be considered a secondary infection without contribution to disease pathogenesis [51]. This quantitative evaluation is in contrast with previous results obtained by van der Hoek and colleagues describing a significantly lower HCoV-NL63 viral load in patients coinfected with RSV or PIV3 than in patients infected with HCoV-NL63 alone [59]. However, the prolonged persistence of HCoV-NL63 at low levels, the different time of sampling relative to the time of disease onset, or the use of different diagnostic technologies could have affected these evaluations (Table 1).

### Bocavirus

Human Bocavirus (HBoV) was discovered in 2005, in Sweden by Allander and colleagues by using a large-scale molecular viral screening technique including DNase sequence-independent single-primer amplification [6]. Since initial observations, several studies have reported the prevalence of human Bocavirus infection all over the world ranging from 2 to 21.5%, mainly in children younger than 3 years of age where it has been associated with upper and with lower RTIs [64-69]. In a study from Norway, HBoV was detected in 12% of children with RTI and it was the fourth most common virus after RSV, HRV and hMPV [70]. Recently, in children with radiographically confirmed community acquired pneumonia in which 17 respiratory viruses were tested during the acute phase of the disease, HBoV was the most frequently detected virus after RSV and HRV [71]. Since the discovery of the first HBoV (HBoV1), three other related bocaviruses (HBoV2, 3 and 4) have been identified in stool samples and associated with gastrointestinal diseases [72,73]. Serological studies on HBoV1 are in line with molecular data.

Serological studies have shown that the mere presence of HBoV DNA in the respiratory tract is not proof of an acute primary infection [65,74-76]. These data are also supported by studies on consecutive respiratory samples showing that HBoV DNA can persist for several months in the respiratory tract [77-79]. Prolonged viral shedding could explain both data reported in some papers in which HBoV DNA was found more often in asymptomatic than symptomatic cases [79,80] and the high percentage of co-infections. In fact, HBoV infections tend to be associated with high rates of coinfections with other viral pathogens such as HRV, adenoviruses, RSV, as well as with bacteria such as *Streptococcus* spp and *Mycoplasma pneumoniae*[46,54,68-70,77,81-83]. Characteristics of persistence and high frequency of coinfections have led to a debate over its role as a true pathogen [84]. Our current knowledge of HBoV infection suggests that the virus is sometimes a passenger and sometimes a pathogen in acute respiratory tract disease and that diagnosis should not be solely based on qualitative PCR in respiratory samples. Indeed, in many studies a positive correlation was seen between respiratory illness and high copy numbers of HBoV1 DNA or the presence of HBoV1 monoinfection [68,75,85-87]. A study performed by Allander and colleagues suggests that acute HBoV infections are associated with the presence of viral DNA in the blood of patients. In fact, HBoV DNA was reported more frequently in patient blood during the acute symptoms than after recovery [68]. In addition, high load and viremic HBoV infection were associated with respiratory tract symptoms, while detection of a low viral load in the nasopharinx alone resulted to have no clinical relevance [68]. Other studies confirmed that HBoV is the most probable cause of respiratory tract disease if the patient has a single infection and high viral load in NPA and viremia [65,70]. However, despite these diagnostic challenges it is becoming increasingly evident that HBoV1 is an important respiratory pathogen [88]. Severe and life-threatening disease has been recently well documented in a 8-month-old child with acute respiratory distress attending an emergency department in Germany [89]. Don et colleagues [76] found serological evidence of an acute HBoV infection in 12% of children with pneumonia and in more than half of these cases with single HBoV infection. In most cases a significant rise in IgG antibodies between paired sera was found in children admitted to hospital for radiologically confirmed pneumonia. IgM antibodies were also detected in all but one patient. This study suggests that HBoV may be a fairly common cause of pneumonia in children Table 1.

### KI and WU Polyomaviruses

Two new polyomaviruses were identified in 2007 in respiratory tract samples following large scale molecular screening using high throughput DNA sequencing of random clones [5,7] and have been named after the institutes where they were found: KI (Karolinska Institute) polyomavirus (KIPyV) and WU (Washington University) polyomavirus (WUPyV). Data on seroprevalence indicate that infection is widespread ranging from 54.1 and 67% for KI and from 66.4% and 89% for WU in North American and German blood donors [90,91]. Since their first identification, KI and WU viral sequences have been confirmed worldwide in respiratory samples from children with respiratory tract disease ranging from 0.2% to 2.7% and from 1.1 to 7%, respectively [91-97]. However WUPyV and KIPyV were found at similar frequencies in control groups without respiratory diseases so the link between these polyomaviruses and acute respiratory diseases remains speculative [94,96,98].

Careful analysis is complicated by high co-infection rates with other well-characterized viral respiratory pathogens. A co-detection rate of 74% has been observed for KIPyV and rates ranging from 68 to 79% for WUPyV [94,95,97]. Therefore, in a recent study in Southern China, hospitalized children with WUPyV infection displayed predominantly cough, moderate fever, and wheezing, but were also diagnosed with pneumonia, bronchiolitis, upper respiratory tract infections and bronchitis. As in most of infected children a single WUPyV infection was detected, it was suggested that the newly described polyomavirus can cause acute respiratory tract infection with atypical symptoms, including severe complications [99]. Nevertheless these data have to be confirmed in further studies.

The presence of WUPyV and KIPyV in samples from children but not from immunocompetent adults suffering from LRTIs suggests that these viruses primarily infect the young population [100]. A correlation between immunosuppression and reactivation of the two novel polyomaviruses has been suggested in immunocompromised patients [101] and in AIDS patients at the molecular level [102], but no evidence of a role of these viruses as opportunistic pathogens has been given.

Overall, these data support the hypothesis that, in analogy with BK and JC polyomaviruses, KIPyV and WUPyV can establish persistent infection, and that virus replication may increase in immunocompromised hosts. However, in a recent study on immunocompetent and immunocompromised adult patients, real-time PCR detected KIPyV and WUPyV in 2.6% and 4.6% of HIV-1–infected patients respectively and in 3.1% and 0.8% of blood donors respectively, while no association was found between CD4+ cell counts in HIV-1 positive patients and infection with KIPyV or WUPyV [103].

KIPyV and WUPyV are also incidentally detected in adults with community acquired pneumonia, in immunocompromised hosts, and in patients with lung cancer; they are more often found in patients suffering an underlying medical condition and coinfections with KIPyV and WUPyV with other respiratory viruses are common [92,103,104]. A recent study evaluating the prevalence and viral load of WUPyV and KIPyV in respiratory samples from immunocompromised and immunocompetent children showed that the prevalence of WUPyV and KIPyV is similar in hematology/oncology patients compared with that of the general pediatric population [105]. High co-detection rates with other respiratory viruses, mainly RSV and enterovirus or rhinovirus, were found for WUPyV and KIPyV in both groups, in analogy with previous reports. However, higher viral loads for KIPyV in the immunocompromised group were detected, suggesting that there may be an increased replication of this virus in this population.

A similar association was not observed for WUPyV. Furthermore, in the immunocompromised group, infection with either virus occurred in older children compared with controls, which may indicate viral-reactivation Table 1.

### Rhinovirus, Enterovirus and Parechovirus

#### Rhinoviruses

HRVs are currently classified in the Picornaviridae family, genus Enterovirus, that includes 3 species: HRV-A, HRV-B, and HRV-C. Within each species there are multiple HRVs designated as “serotypes”, “types”, or “strains”. Several recent epidemiological studies suggest that HRV-A and HRV-C are the predominant species associated with acute respiratory illnesses in hospitalized children and adults, compared to HRV-B which are rarely detected [106].

The new HRV lineage designated HRV-C has been identified using molecular methods and associated with severe clinical presentations in infants and immunocompromised adults. Symptoms of patients infected with this new strain were mainly bronchiolitis, wheezing, and asthmatic exacerbation in cases from Australia and Hong Kong, which peaked in fall and winter whereas in New York the new rhinovirus genotype was detected in cases of influenza like illness (ILI) that were clustered within an 8-week period from October to December [62,107,108]. A recent study describes a clinical case of severe respiratory and pericardial disease in an infant infected by HRV-C suggesting tha viral tropism is not strictly restricted to the respiratory tract [109]. A study focusing on the global distribution of novel rhinovirus indicates its association with community outbreaks and pediatric respiratory disease also in Africa and in symptomatic subjects living in remote locations having limited contacts with other human populations. Moreover evidence for a role of HRV-C in lower respiratory tract disease and febrile wheeze in infants and asthma exacerbations in older children was reported [110-114]. Recent studies making comparisons between HRVs species, found the HRV Cs more so than As or Bs as the major contributors to febrile wheeze and asthma exacerbation in infants and children, respectively . However, the severity of clinical manifestations for HRV-C is comparable to that for HRV-A in children with community-acquired pneumonia [115]. In HRV C studies so far, no clear clinical difference has been noted between patients with single or mixed HRV-C infection [111,113,116]. In a study, monoinfection was observed in more than half of cases and was more common than RSV monoinfection in patients with upper RTD, however the duration of hospitalizations was not significantly different between the HRV-C monoinfection group, HRV-A or HRV-B monoinfection group and RSV group suggesting that HRV-C is an important etiological factor in children with RTI [117]. Most HRV-C co-detections are with RSV [113,117-119], however in a large study HRVs were statistically the least likely virus of 17 examined to be associated with co-infections [120] Table 1.

### Enteroviruses

A novel HEV (EV104) genotype was first identified in Switzerland in 2010 from respiratory samples collected during 2004–2007 in 8 children with respiratory signs and symptoms and acute otitis media [121]. In a following epidemiologic study conducted in Italy, five strains of the new EV104 genotype were detected in patients with respiratory diseases [122]. Patients were aged 2 to 62 years and most had underlying diseases; in immunocompetent patients EV104 was associated with chronic rhinopharyngytis, whereas in immunocompromised patients with symptoms of acute respiratory tract infection including wheezing, fever and rhinorrhea. Only in one out of 5 patients a coinfecting virus was detected.

EV109 was first discovered from a case of acute respiratory illness in a Nicaraguan child in September 2010; EV109 isolates were then detected in 1.6% of respiratory samples of children with influenza like illness (ILI) in Managua, and recognized to have a pathogenetic role in the illness [2]. After this characterization, a species C of HEV distantly related to EV109 was retrieved from a rectal swab of a deceased patient during an outbreak of flaccid paralysis in Congo; in this sample poliovirus and other neurologic, enteric and respiratory viral pathogens were not detected [123]. The global distribution of EV 109 is currently unknown and only a few studies have been performed yet to evaluate epidemiological features of this infection. [124,125] To date the pathogenic role of these new enterovirus strains has still to be defined in larger clinical and epidemiological surveys Table 1.

### Parechoviruses

There are fourteen known HPeV genotypes that were isolated mainly in young children [126-134]

Two recent studies have investigated the involvement of HPeVs in respiratory diseases reporting a low frequency of detection and a lack of clear disease association [135,136]. Both studies detected HPeV infections mostly in children below 5 years and the most commonly identified parechoviruses were HPeV1 and HPeV3, while HPeV4, HPeV 5 and HPeV 6 were detected in a minority of cases. However, in addition to a low HPeVs prevalence in respiratory samples, a high rate of co-infection with other respiratory viruses was observed in HPeV positive samples, making it difficult to define a pathogenic role of these new HPeV genotypes in child respiratory infections.

### Pandemic A/H1N1v influenza 2009

In March 2009, a novel reassortant influenza strain (A/H1N1v) was discovered in Mexico as an infective agent in humans [137,138]. From April 15 through May 5, 2009, a total of 642 confirmed cases of human infection with the outbreak strain of H1N1v were identified in 41 states in the USA [139,140]. Of the patients with confirmed infection, 9% required hospitalization. The age of hospitalized patients ranged from 19 months to 51 years and among the hospitalized patients for whom data were available, 18% were children under the age of 5 years. Multiple reports have described the clinical features of infection with this novel virus, which are similar or milder to those of seasonal influenza, [141,142]. The spectrum of clinical presentation varies from self limiting respiratory tract illness in most cases, to primary viral pneumonia resulting in respiratory failure, acute respiratory distress, multi-organ failure and death, most of them occurring in patients with underlying medical conditions [142].

During the A/H1N1v flu wave, due mainly to the lack of cross-neutralizing anti-influenza antibodies [143,144] or the presence of co-morbidities, more children and younger adults were infected by the pandemic flu strain and had serious disease than in the seasonal epidemic. For this reason they were identified as a particular risk group, together with pregnant women [145,146]. Young children presented a higher attack rate than older adults and a greater mortality rate than previously observed with classical seasonal flu [147,148]. In a study conducted in England from June 2009 to March 2010, a childhood mortality rate of 6 per million population was reported. The rate was highest for children less than 1 year and with severe pre-existing disorders [149]. Similar data are reported in other studies highlighting that most severe cases occurred in children with known comorbidities [150-153].

The occurrence of co-infection with influenza A H1N1v and other respiratory viruses has been reported in few studies. In a study conducted in France and aimed at investigating respiratory pathogens involved in ILI during the early weeks of the 2009–29010 H1N1v diffusion, 19% of samples positive for H1N1v were also positive for other respiratory pathogens. In mixed infections, HRV was the more frequent co-pathogen being detected in 13.2% of all samples positive for H1N1v [154]. However, HRV infections represented nearly half of non-H1N1v viral infections and the increase of H1N1v positive cases was associated with a rapid decline of HRV infections. In addition, the frequency of virus co-infection was slightly but not significantly higher in samples positive for H1N1v as compared with samples positive for other respiratory pathogens and viral mixed infection both with H1N1v and other viruses was not associated with a different clinical presentation. Similar co-infection data are reported in a prospective study evaluating a combination of two RT-PCR DNA microarray systems in virological routine diagnosis of ILI [155]. In both studies conducted in France, in the same H1N1v early pandemic period, few RSV infections were reported compared with epidemiological data in the same period of the last four years. The delayed and reduced circulation of RSV observed in 2009–10 compared with 2008–09 suggests that the early circulation of the 2009 pandemic influenza A(H1N1) viruses had an impact on the RSV epidemic [156]. A significant inverse relationship between HRV and A H1N1 pandemic virus was also reported, suggesting that the presence of HRV reduces the risk of infection by the H1N1 virus and thus, indirectly, the spread of the virus [156] Table 1.

### Orphan viruses

Torque tenovirus (TTV), is classified in the new genus *Anellovirus*[157]. TTV produces long-lasting (possibly life-long) viremia in about 80% of apparently healthy individuals of all ages and living in different countries[158]. Although no evidence was obtained that TTV is a direct cause of acute respiratory diseases, it was observed that average viral loads are considerably higher in children with bronchopneumonia than in children with milder illnesses, regardless of the presence of common respiratory viruses [159]. Further studies could not confirm the association, but documented a link between TTV infection in children and asthma, suggesting that TTV might be a contributing factor in the lung impairment caused by this condition [160]. Finally, it was documented that TTV is able to infect respiratory ciliated cells and that these cells are potentially able to support viral replication [161]. Far from being conclusive, the data suggest that these viruses may replicate efficiently in the respiratory tract of children with and without acute respiratory infections by other respiratory viruses, and that although TTV does not have a clear pathogenic role in acute respiratory diseases, it may influence the clinical presentation of the disease.

## Conclusions

Over the past 10 years, advances in scientific knowledge and the availability of new technologies have deeply changed our views in the field of respiratory viral infections. In particular, the identification of new viruses or viral strains has allowed to better define the viral etiology in many respiratory diseases where the viral pathogen was only hypothesized or suspected.

Molecular virological diagnosis has thus gained an increasing importance with the development of novel molecular assays, in some cases able to detect many respiratory viruses simultaneously.

Nevertheless, despite the availability of novel diagnostic assays, data are still controversial regarding the role of coinfection in a more severe clinical outcome in comparison to single infections.

In this context, even though specific antiviral compounds or vaccines are being developed, the level of accuracy and the associated higher costs of complex molecular assays to highlight the presence of one or more viruses in the same samples, are often considered not to be clinically necessary.

However, some important considerations emphasize the importance of implementing diagnostic approaches that allow the identification of the greatest number of newly identified or emerging viral respiratory pathogens. Firstly, the use of diagnostic assays able to simultaneously detect more respiratory viruses, including the newly identified ones, and in some cases their load, will help to clarify virus-host interactions which are still partially unknown, in particular in hospitalized patients. This will allow to develop appropriate control measures for nosocomial infection containment. Moreover, in many cases respiratory viruses pave the way to severe secondary bacterial infections. Thus the rapid identification of viral pathogens may help to limit disease progression and to plan appropriate monitoring and patient management in defined clinical settings. Furthermore, rapid and reliable screening with large panel of respiratory viruses responsible for upper and lower RTIs is of major epidemiological and clinical interest for monitoring influenza pandemic waves or unexpected respiratory outbreaks.

In addition, in the case of emerging viral strains as well as for enteroviruses, parechovirus or rhinoviruses the subsequent or simultaneous circulation of genetically distinct strains with distinct pathogenic potential, suggests a high risk for repeated pediatric respiratory infections as well as the possibility of genetic recombination within species. In these cases, the continuous development of quantitative assays associated with viral genotyping assays will allow the rapid and valuable etiological diagnosis of enterovirus, parechovirus or rhinovirus childhood infections, helping to prevent both nosocomial transmission and to control the emergence of new respiratory strains with unpredictable pathogenic potential.

## Competing interests

The authors declare that they have no competing interests.

## Authors’ contributions

MD, FC, MC wrote the manuscript. ERC organized the table. All authors read and approved the final manuscript.

## Authors’ information

**Maurizia Debiaggi** graduated with a first-class degree in Biological Sciences at the University of Pavia in 1984. From 1984 to 1991, following the award of a Research Grant in the area of infectious diseases, she worked in the Department of Microbiology, University of Pavia. In these years she acquired skills in molecular virology and diagnostic microbiology. Since November 1992 she is Researcher at the Department of Clinical and Diagnostic Sciences, Unit of Microbiology, University of Pavia. Her scientific activity has been mainly intent on molecular virology studies; she focused on the molecular epidemiology of emerging viruses such as the novel Human Metapneumovirus, NL-63 and HKU1 coronaviruses and the human Bocavirus. Her teaching activity is currently held in the official courses of Medical Microbiology for medical students as well as for students obtaining their Degree in Medical Biothecnologies at the Medical School of the University of Pavia.

**Filippo Canducci** graduated in Medicine Cum Laude (Universtà Cattolica, Roma) in 2001. In 2005 he became PhD in Pharmacology, Chemotherapy and Microbiology (University of Trieste). In 2008 he specialized in Microbiology and Virology at the Università Vita-Salute San Raffaele where since 2009 he is Contract Professor in Microbiology. In 2003, he isolated and characterized the SARS Coronavirus strain HSR1 and in 2004 he was awarded by the Carlo Urbani Foundation. Dr Canducci has studied the prevalence and variability of emerging respiratory viruses such as the human Metapneumovirus, the Coronvaviruses NL-63 and HKU1, human Bocavirus and pandemic Influenza A virus. Since 2003 he has studied HIV-1 infection in vitro in vivo by original phenotypic assays. Dr Canducci is now also leading a research group to study the pathogenetic mechanisms of atherosclerosis. In 2010 Dr Canducci was granted the Young Investigators Grant from the Italian Ministry of Health.

**Elisa Rita Ceresola** graduated cum laude in Biological Sciences at the Università Vita-Salute San Raffaele in 2009. She is currently specializing in Microbiology and Virology in the same University. Elisa Rita Ceresola has focused her research on the epidemiology and molecular variability of emerging respiratory viruses and on optimization of molecular assays to characterize HIV-1 infection in vitro and ex-vivo.

**Massimo Clementi** is Dean of the Faculty of Medicine and Professor of Microbiology and Virology at the Università Vita-Salute San Raffaele. He is chief of the Laboratory of Microbiology and Virology, Diagnostica e Ricerca San Raffaele, Milan, Italy. He has research interests in the virus-host interplay in the course human immunodeficiency virus and of hepatitis C virus infections, in the microbiology of emerging respiratory infectious diseases, and in infections of immunocompromised patients.
